# Supplementary material for: BETs inhibition attenuates oxidative stress and preserves muscle integrity in Duchenne muscular dystrophy
Source: Nat Commun. 2020 Nov 30;11:6108. doi: 10.1038/s41467-020-19839-x (PMC7705749; doi:10.1038/s41467-020-19839-x)
Supplement: Supplementary file 1 — Supplementary Information [file 41467_2020_19839_MOESM1_ESM.pdf]

BETs inhibition attenuates oxidative stress and preserves muscle integrity in Duchenne Muscular Dystrophy.

Marco Segatto[1,2,#] Roberta Szokoll[1,#], Raffaella Fittipaldi[1], Cinzia Bottino[1], Lorenzo Nevi[1], Kamel Mamchaoui[3], Panagis Filippakopoulos[4] and Giuseppina Caretti[1,*].

Supplementary materials:

-Supplementary figures

-Supplementary tables

Supplementary Figures

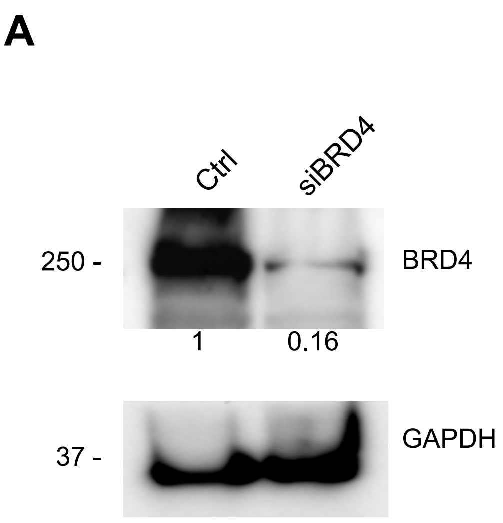

Supplementary Figure 1. Validation of BRD4 antibody.

BRD4 antibody was tested in immunoblot with extracts of C2C12 cells transfected with siRNAs targeting BRD4 transcript and a scramble siRNA. Normalized band intensity of BRD4 is reported below signals.

A

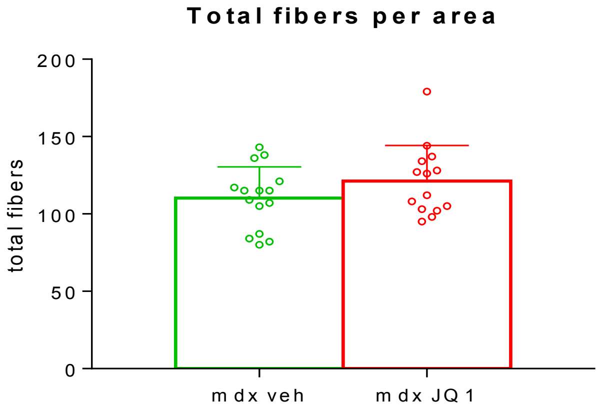

B

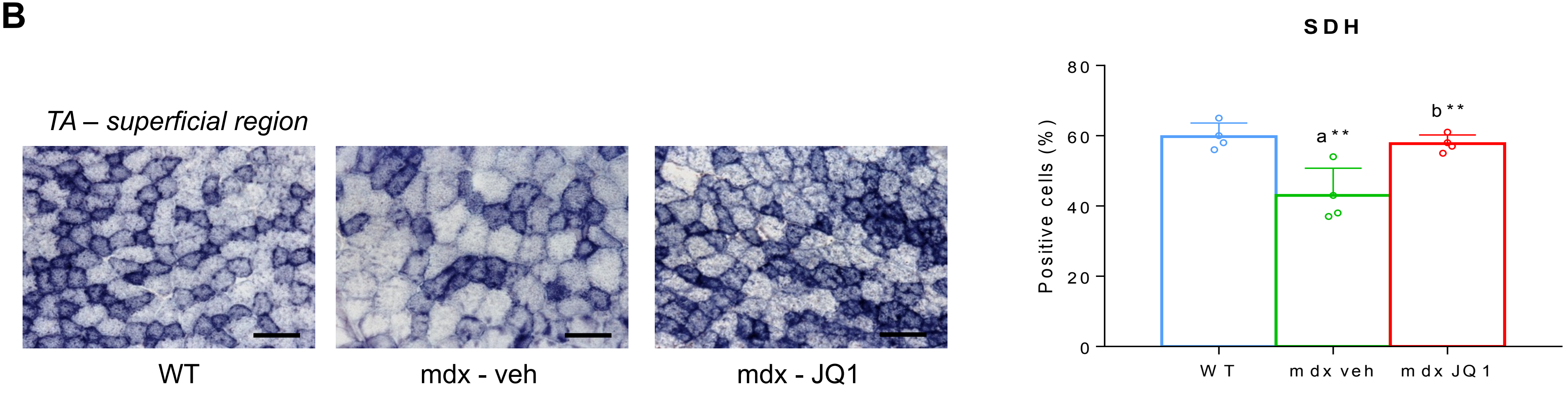

Supplementary Fig.2

Supplementary Figure 2. SDH staining increases following JQ1 treatment.

(A) Total fiber per area was not altered by JQ1 treatment. The total number of fibers was counted in transversal muscle sections. Data represent mean±SD. Mdx veh: n=15 sections were examined from 3 animals; mdx JQ1: n=14 sections were examined from 3 animals. Statistical significance was determined by using unpaired t-test.

(B) SDH staining from muscle superficial region of TAs, from control, vehicle– and JQ1-treated mice. Scale bar: 50 μm. Right panel: quantification of SDH staining intensity. Statistical significance was determined by using one-way ANOVA followed by Tukey's post hoc test. n=4. ** $P < 0.01$.

"a" indicates statistical significance compared to Control group; "b" indicates statistical significance compared to the "mdx mice" animal group.

A

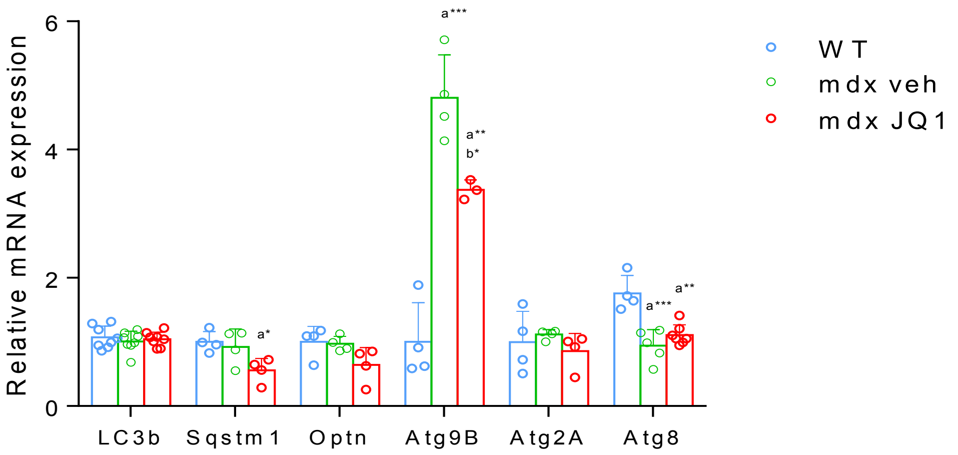

Supplementary Figure 3. JQ1 does not increase autophagy gene transcription.

(**A**) qRT-PCR analysis of transcript levels for LC3b (n=8 for each experimental group), Sqstm1 (n=4 for each experimental group), Optn (n=4 for each experimental group), Atg9B (WT, n=4; mdx, n=4; mdx+JQ1, n=3), Atg2A (n=4 for each group), and Atg8 (WT, n=4; mdx, n=5; mdx+JQ1, n=7) in TAs from wild type, vehicle-, JQ1-treated mice. Data are normalized against HPRT and expressed as the mean±SD. * $P < 0.05$; ** $P < 0.01$; *** $P < 0.001$. Statistical significance was determined by using one-way ANOVA followed by Tukey's post hoc test.

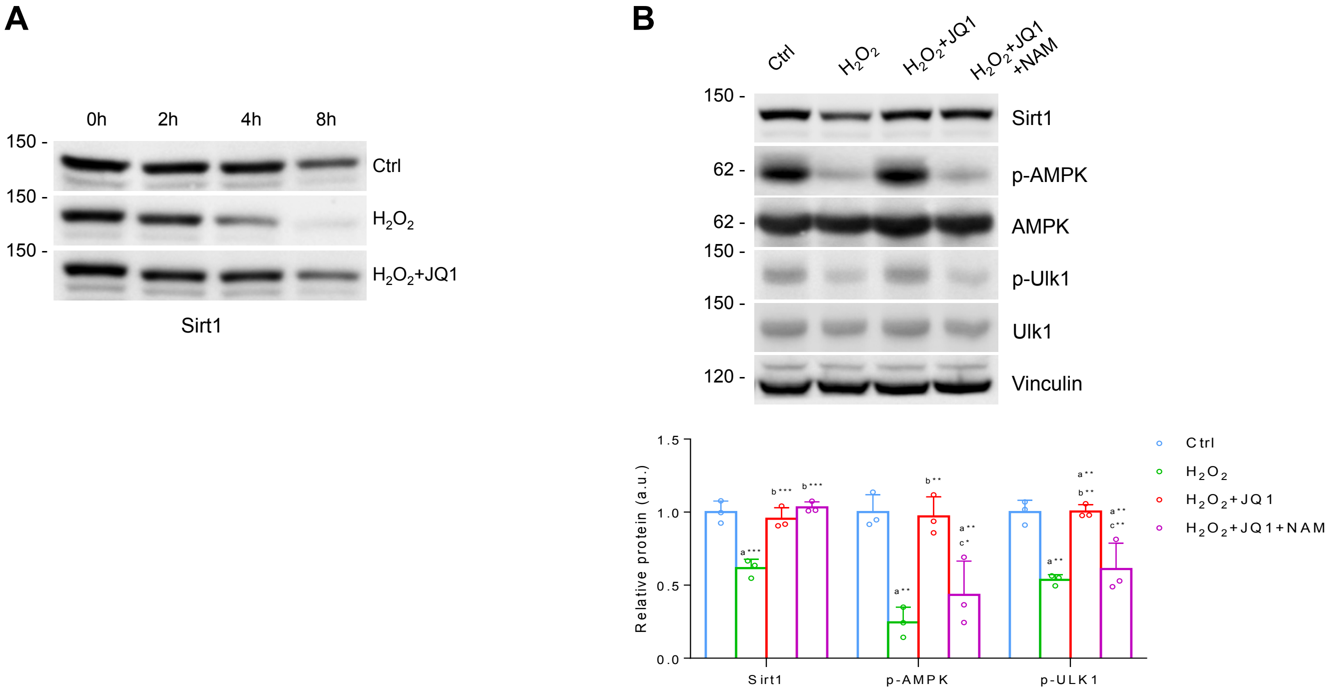

Supplementary Figure 4. Rescue in AMPK activation requires Sirt1 activity.

(**A**) Representative immunoblot and densitometric analysis of Sirt1 degradation assay performed on H_2O_2-treated C2C12 cells treated with H_2O_2 (250μM) alone or in combination with JQ1 (0.2μM). n=2.

(**B**) Representative images of immunoblot for Sirt1, p-AMPK, AMPK, Ulk1, p-Ulk1 (Ser555), in C2C12 myoblast extracts of control and H_2O_2-, H_2O_2/JQ1- and H_2O_2/JQ1/NAM co-treated cells. Data represent mean±SD, n=3 independent experiments. Statistical significance was determined by using one-way ANOVA followed by Tukey's post hoc test. * $P < 0.05$; ** $P < 0.01$; *** $P < 0.001$. "a" indicates statistical significance compared to vehicle-treated C2C12 myotubes (Ctrl); "b" indicates statistical significance compared to H_2O_2-treated cells, "c" indicates statistical significance compared to H_2O_2+JQ1-treated C2C12.

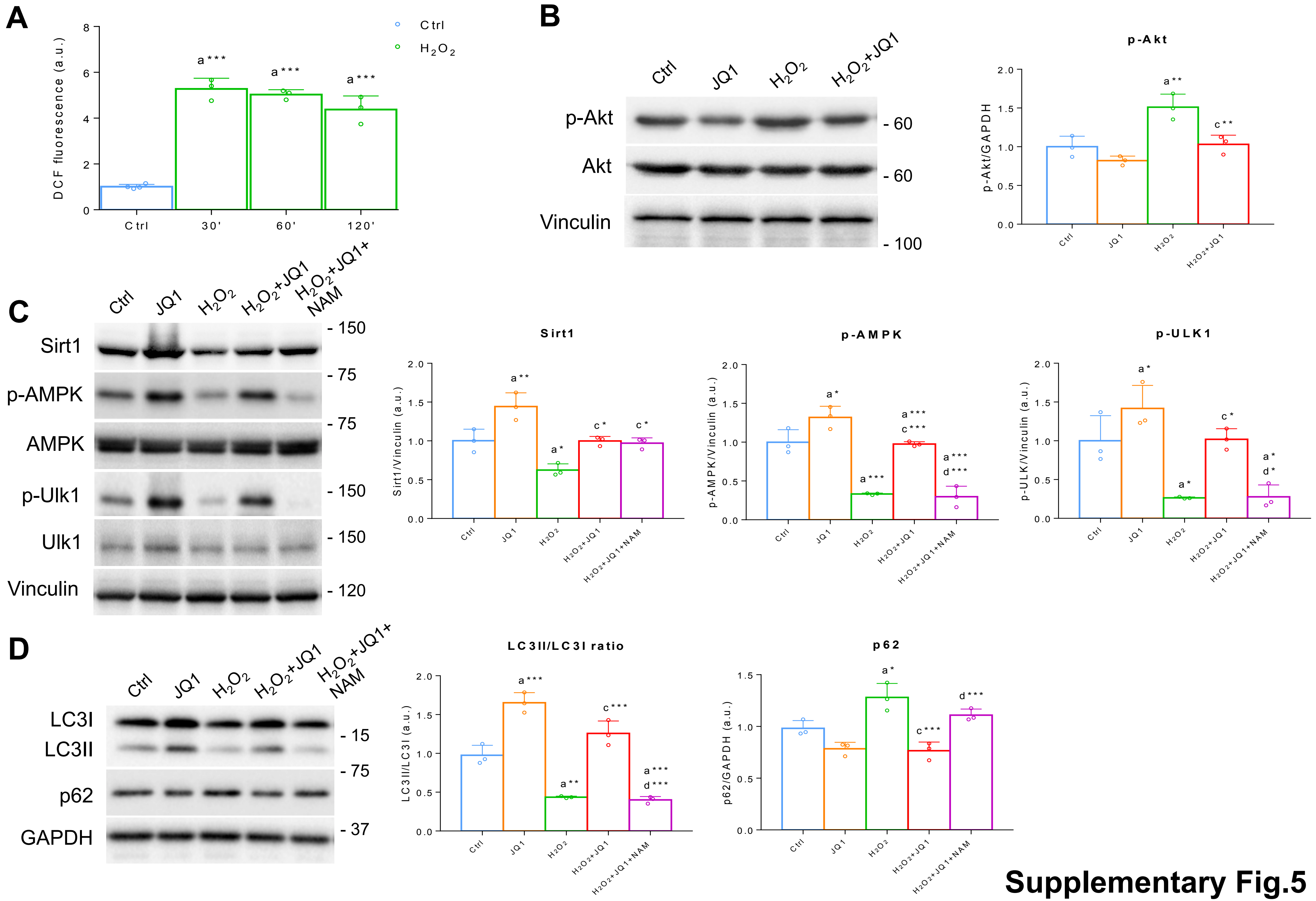

Supplementary Fig.5

Supplementary Figure 5. JQ1 tempers oxidative stress in C2C12 myoblasts, when oxidative stress is pre-existent.

(A) DCF fluorescence was measured after 30-60-120 minutes of H_2O_2 (250μM) stimulation in C2C12 myoblasts. Ctrl: n=4; H_2O_2: n=3. Data represent mean±SD. ; *** represents $P < 0.001$.

(**B**) Representative western blot for total and p-AKT in C2C12 myotube extracts of control, JQ1-, H_2O_2- and H_2O_2/JQ1-treated cells. Myotubes were treated with H_2O_2 (250μM) for 2 hr and then JQ1(200nM) was added for 24 hr. Vinculin serves as a loading control. Right panel: quantification of normalized band intensity derived from three independent experiments. Data represent mean±SD, n=3. ; ** denotes $P < 0.01$. “a” indicates statistical significance compared to Control cells; “c” indicates statistical significance compared to the H_2O_2-treated C2C12.

(**C**) Representative western blot for Sirt1, p-AMPK, AMPK, p-Ulk1 (Ser555), Ulk in C2C12 myoblast extracts of control, JQ1-, H_2O_2-, H_2O_2/JQ1-, and H_2O_2/JQ1/NAM treated cells. Myotubes were treated with H_2O_2 (250μM) for 2 hr, and then JQ1 (200nM) or JQ1/NAM was added for 24 hr. Vinculin serves as a loading control. Right panel: quantification of normalized band intensity derived from n=3 independent experiments. Data represent means ± SD. Statistical analysis was assessed by using one-way ANOVA followed by Tukey’s post hoc test. *$P < 0.05$; ** $P < 0.01$; *** $P < 0.001$. “a” indicates statistical significance compared to Control cells; “b” indicates statistical significance compared to the JQ1-treated C2C12; “c” indicates statistical significance compared to the H_2O_2-treated C2C12; “d” indicates statistical significance compared to the H_2O_2+JQ1-treated C2C12.

(**D**) Representative western blot for LC3I, LC3II and p62 in C2C12 myoblast extracts of control, JQ1-, H_2O_2-, H_2O_2/JQ1-, and H_2O_2/JQ1/NAM treated cells. Myotubes were treated with 250μM H_2O_2 for 2 hr, then 200nM JQ1 or JQ1/NAM was added for 24 hr. In order to study the autophagy flux, the experiment was performed pre-treating cells with 30 μM of the lysosomotropic agent chloroquine. GAPDH serves as a loading control. Right panel: quantification of normalized band intensity derived from 3 independent experiments. Data represent means ± SD. Statistical analysis was assessed by using one-way ANOVA followed by Tukey's post hoc test. *$P < 0.05$; ** $P < 0.01$; *** $P < 0.001$. "a" indicates statistical significance compared to Control cells; "b" indicates statistical significance compared to the JQ1-treated C2C12; "c" indicates statistical significance compared to the H_2O_2-treated C2C12; "d" indicates statistical significance compared to the H_2O_2+JQ1-treated C2C12.

A

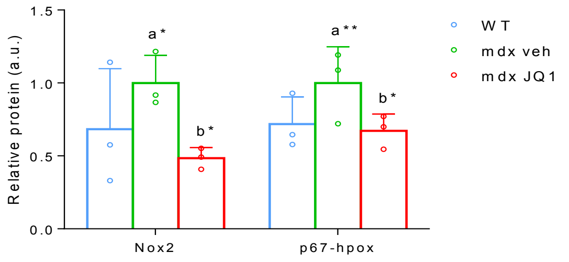

B

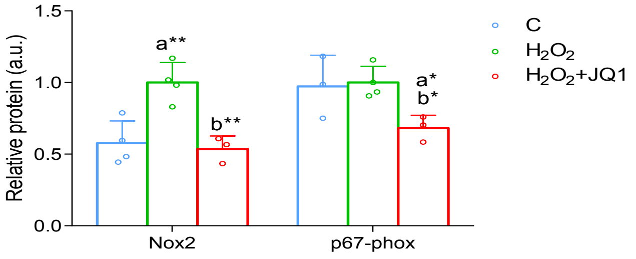

Supplementary Figure 6. Quantification of normalized band intensity presented in Fig. 5B and 5G. Data represent mean±SD. Statistical analysis was assessed by using one-way ANOVA followed by Tukey's post hoc test. * denotes $P < 0.05$; ** $P < 0.01$.

(A) "a" indicates statistical significance compared to WT mice; "b" indicates statistical significance compared to mdx mice.

(B) "a" indicates statistical significance compared to Control cells; "b" indicates statistical significance compared to the H_2O_2-treated C2C12.

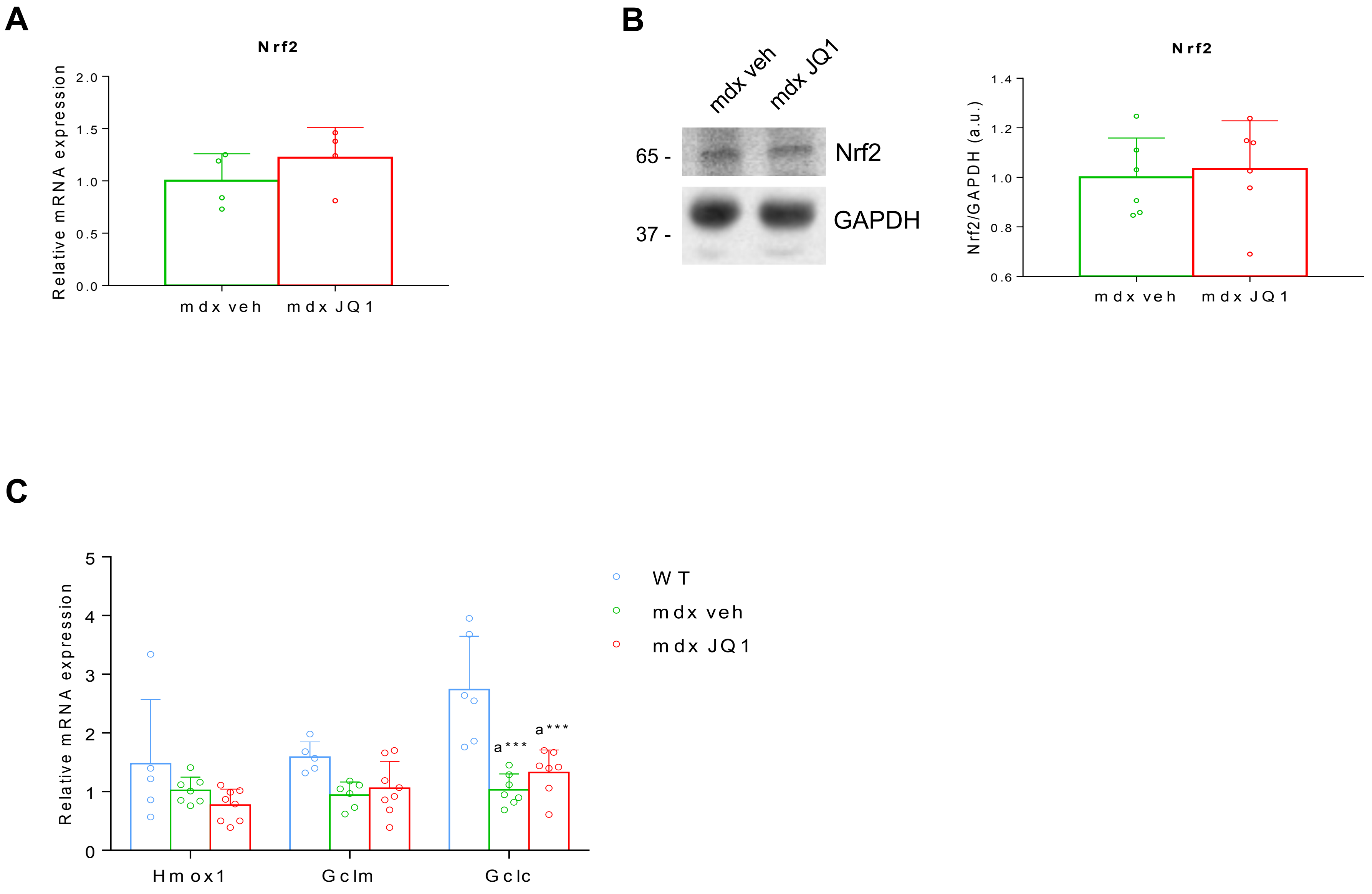

Supplementary Fig.7

Supplementary Figure 7. Antioxidant genes transcription is not activated by JQ1.

(**A**) qRT-PCR analysis of Nrf2 transcript levels was performed in TAs from control, vehicle-, JQ1-treated mice. Data are normalized against HPRT and expressed as the mean±SD, n=4 for mdx mice. Statistical significance was determined by using unpaired t-test.

(**B**) Immunoblot analysis of Nrf2 levels in vehicle- and JQ1-treated mdx mice. Right panel: quantification of normalized band intensity. Data represent means±SD, n=6 animals per group. Statistical significance was determined by using unpaired t-test.

(**C**) qRT-PCR analysis of transcript levels for Hmox1 (WT, n=5; mdx, n=7; mdx+JQ1, n=8), Gclm (WT, n=5; mdx, n=6; mdx+JQ1, n=8), Gclc (WT, n=6; mdx, n=7; mdx+JQ1, n=7) was performed in TAs from control, vehicle-, JQ1-treated mice. Data are normalized against HPRT and expressed as the mean±SD. *** $P < 0.001$. Statistical significance was determined by using one-way ANOVA followed by Tukey's post hoc test. "a" indicates statistical significance compared to control.

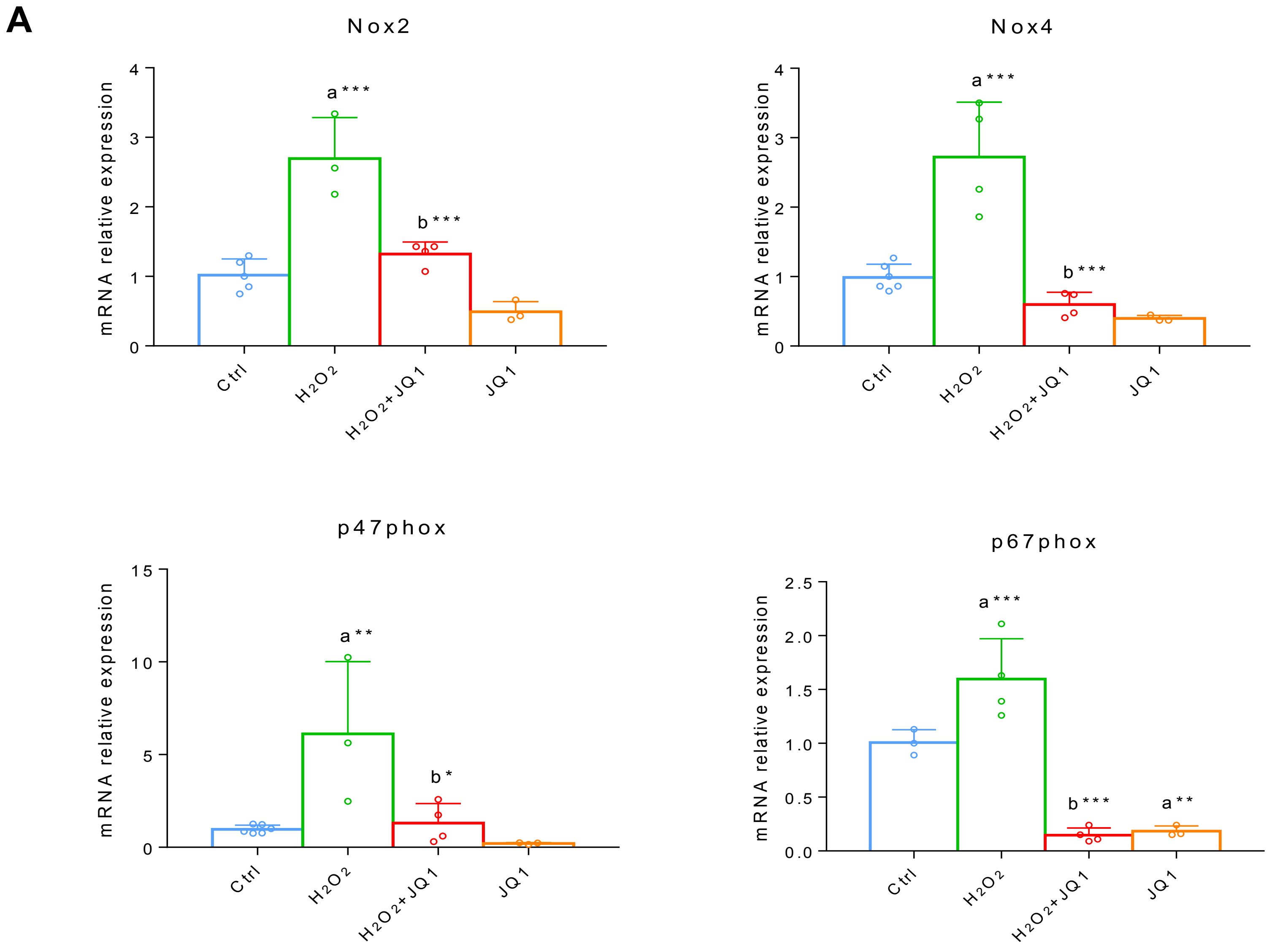

Supplementary Fig.8

Supplementary Figure 8. JQ1 decreases NADPH subunit transcription in C2C12 myoblasts, when oxidative stress is pre-existent.

(**A**) C2C12 myotubes were treated with 250μM H_2O_2 for 2 hr, then 200nM JQ1 was added for 24 hr. RNA was isolated with Trizol and RNA was reversed transcribed. Graphs represent qRT-PCR analysis of transcript levels of Nox2 (Ctrl, n=5; H_2O_2, n=3; H_2O_2+JQ1, n=4; JQ1, n=3), Nox4 (Ctrl, n=6; H_2O_2, n=4; H_2O_2+JQ1, n=4; JQ1, n=3), p47-phox (Ctrl, n=6; H_2O_2, n=3; H_2O_2+JQ1, n=4; JQ1, n=3) and p67-phox (Ctrl, n=3; H_2O_2, n=4; H_2O_2+JQ1, n=4; JQ1, n=3). Data are normalized against HPRT and expressed as the mean±SD. * $P < 0.05$; ** $P < 0.01$; *** $P < 0.001$. Statistical significance was determined by using one-way ANOVA followed by Tukey's post hoc test. "a" indicates statistical significance compared to Control cells; "b" indicates statistical significance compared to the H_2O_2 -treated C2C12.

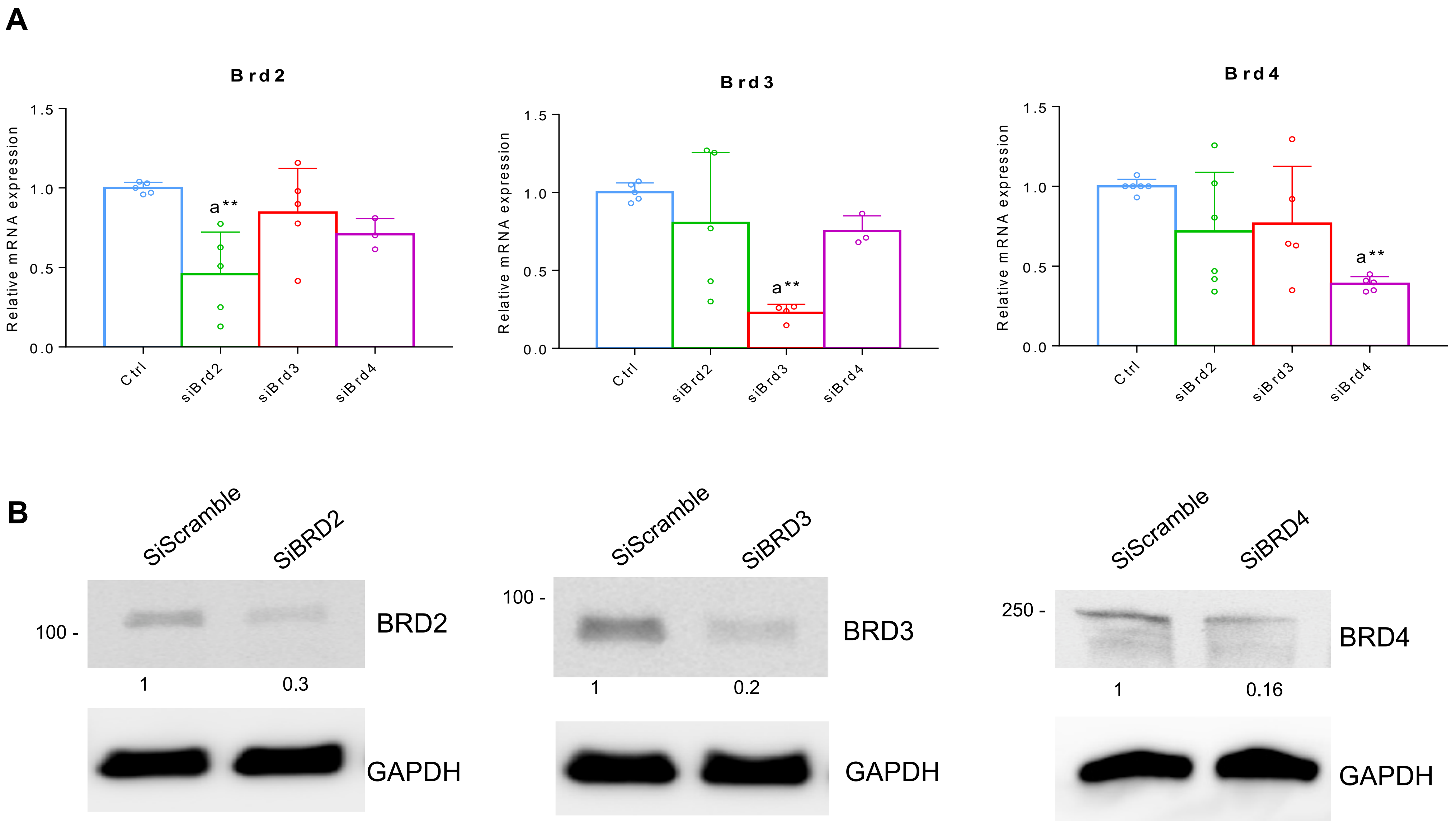

Supplementary Fig.9

Supplementary Figure 9. BET proteins siRNAs.

(A) qRT-PCR analysis of Brd2 (Ctrl, n=5; siBrd2, n=5; siBrd3, n=5; siBrd4, n=3), Brd3 (Ctrl, n=5; siBrd2, n=5; siBrd3, n=4; siBrd4, n=3) and Brd4 (Ctrl, n=6; siBrd2, n=6; siBrd3, n=5; siBrd4, n=5) mRNA levels in C2C12 cells transfected with siRNAs targeting BRD2, BRD3 or BRD4. Data are normalized against GAPDH and expressed as the mean±SD. * $P < 0.05$, ** $P < 0.01$, *** $P < 0.001$. Statistical significance was determined by using one-way ANOVA followed by Tukey's post hoc test. "a" indicates statistical significance compared to scramble-transfected cells.

(B) Immunoblots of C2C12 extracts used in panel A. Normalized band intensity of BRD proteins is reported below signals.

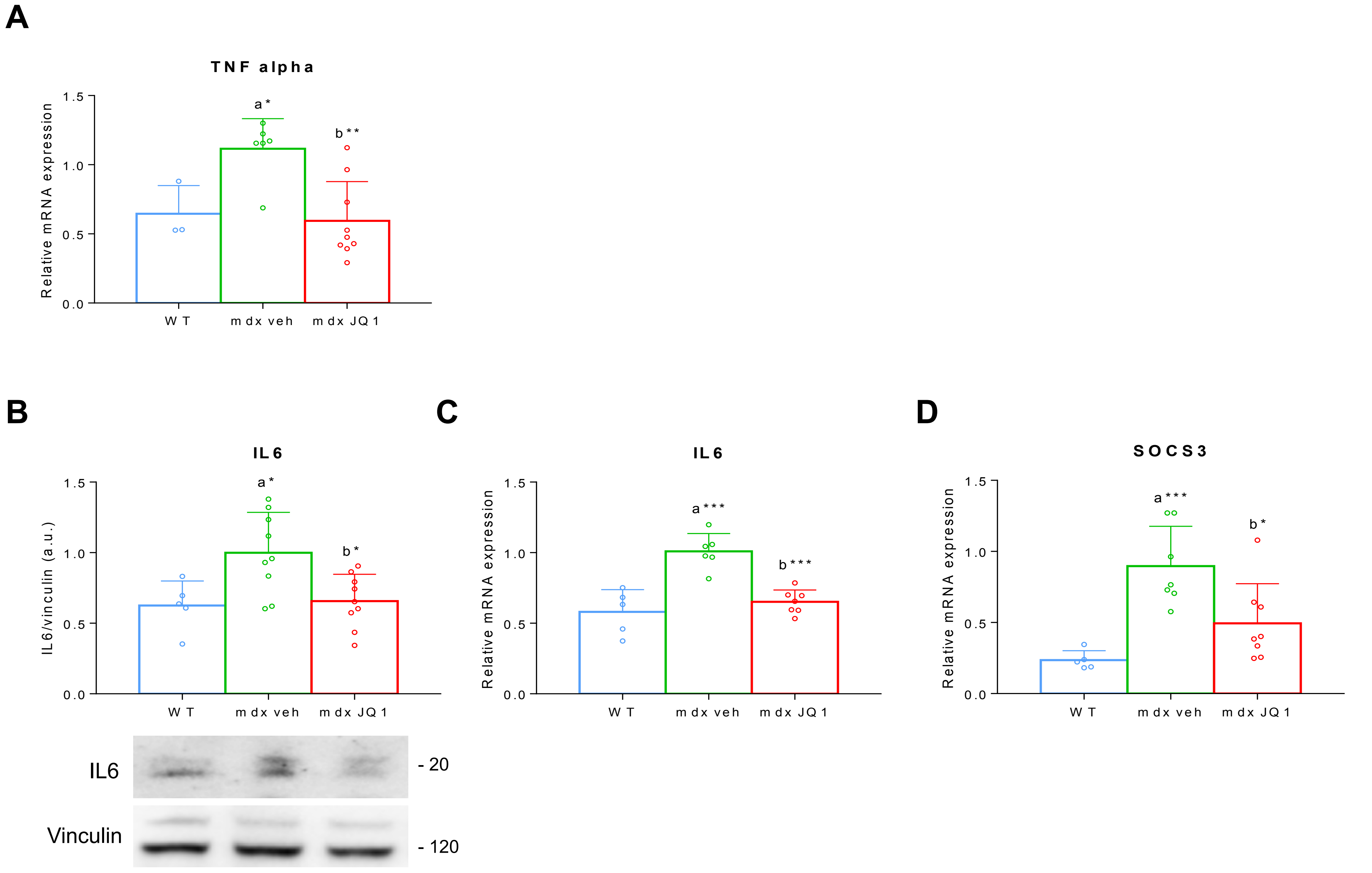

Supplementary Fig.10

Supplementary Figure 10. JQ1 tempers inflammation in the mdx skeletal muscle.

(**A,C,D**) qRT-PCR analysis of TNFalpha (A) (WT, n=3; mdx, n=6; mdx+JQ1, n=9), IL6 (C) (WT, n=5; mdx, n=6; mdx+JQ1, n=7) and SOCS3 (D) (WT, n=5; mdx, n=7; mdx+JQ1, n=8) mRNAs in TAs from wt mice, vehicle-, JQ1-treated mdx mice. Data are normalized against HPRT and expressed as the mean±SD. * $P < 0.05$; ** $P < 0.01$; *** $P < 0.001$. Statistical significance was determined by using one-way ANOVA followed by Tukey's post hoc test.

(**B**) Representative images of immunoblots for IL6 in control (wt mice), vehicle- and JQ1-treated mdx mice.

Vinculin is used as a loading control. Upper panel: signal quantification was performed with ImageJ. Data are expressed as the mean±SD. n=5 (wt experimental group), n=9 (mdx experimental groups), * P <0.05 was determined by using unpaired t-test.

"a" indicates statistical significance compared to control group. "b" indicates statistical significance compared to the "mdx mice" animal group.

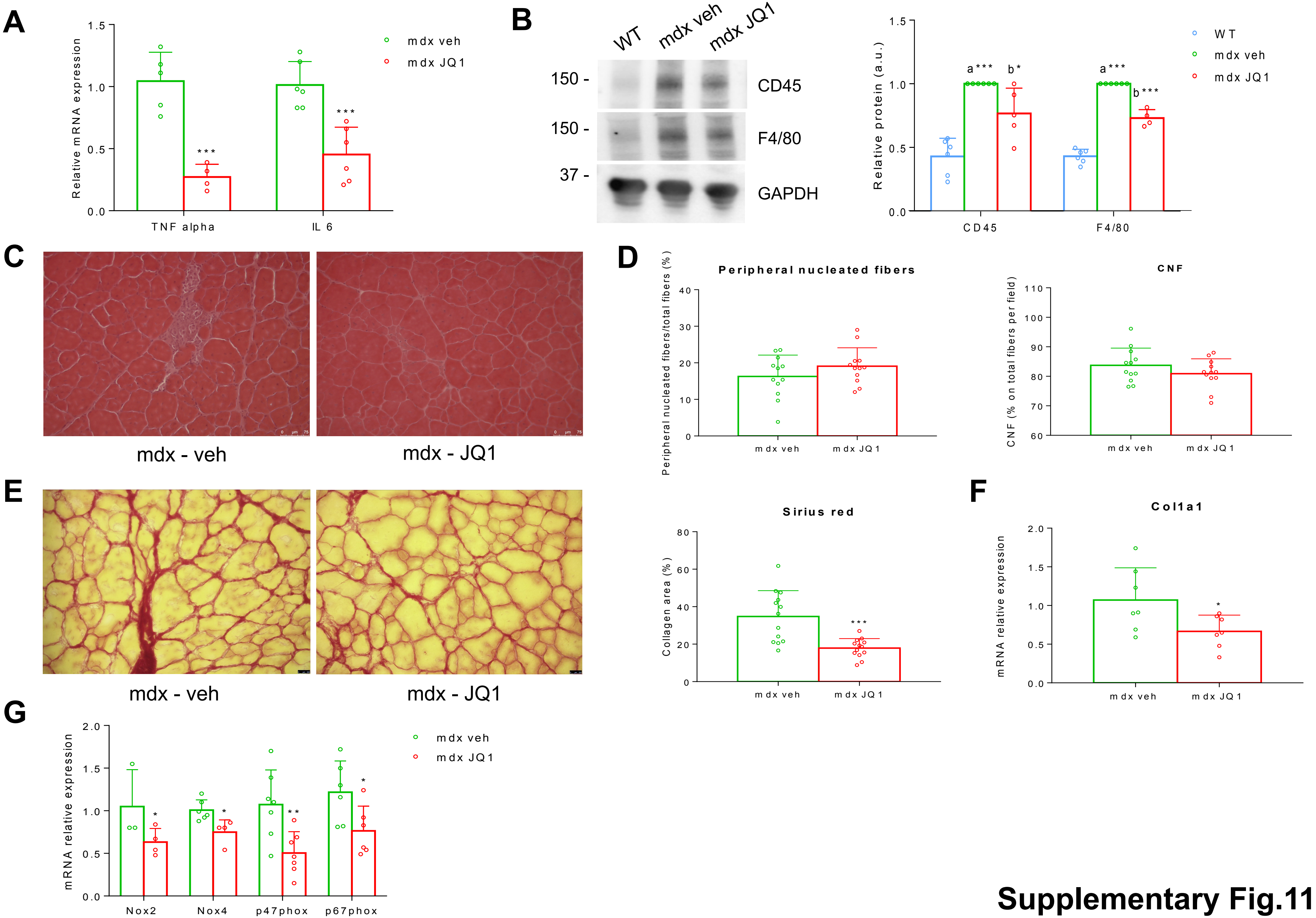

Supplementary Fig.11

Supplementary Figure 11. JQ1 is also beneficial at later stage of disease progression.

(**A**) qRT-PCR analysis of TNFalpha (mdx veh, n=5; mdx JQ1, n=4) and IL6 (mdx veh, n=6; mdx JQ1, n=6) mRNAs in TAs from vehicle- and JQ1-treated 12-month-old mdx mice. Data are normalized against HPRT and expressed as the mean±SD. *** $P < 0.001$. Statistical significance was determined by using one-way ANOVA followed by Tukey's post hoc test.

(**B**) Immunoblot of F4/80 (WT, n=6; mdx, n=6; mdx+JQ1, n=4) and CD45 (WT, n=6; mdx, n=6; mdx+JQ1, n=5) shows attenuation of these markers in TA muscles of 12-month-old mdx mice. Scale bar: 50 μm. Data are expressed as the mean±SD. Statistical significance was determined by using one-way ANOVA followed by Tukey's post hoc test. * denotes P< 0.05, *** denotes $P < 0.001$. Statistical significance was determined by using one-way ANOVA followed by Tukey's post hoc test.

(**C**) Hematoxylin/Eosin staining of TA muscles from JQ1- and vehicle-treated 12-month-old mdx mice (n=3 per experimental group).

(**D**) Percentage of peripheral and centrally nucleated fibers per area were evaluated from Hematoxylin/Eosin staining of TA muscles (n=12 sections were examined from n=3 animals for each experimental group). Scale bar: 50 μm. Statistical significance was determined by using unpaired t-test.

(**E**) Sirius red staining shows attenuation of fibrosis in JQ1-treated 12-month-old mdx mice (n=12 sections were examined from n=3 animals for each experimental group). Scale bar: 50 μm. Right panel: quantification of staining. *** P <0.001. Statistical significance was determined by using unpaired t-test.

(**F**) qRT-PCR analysis of collagen1a1 mRNA in TA muscles from vehicle- and mdx-

treated 12-month-old mdx mice. Data are normalized against HPRT and expressed as the mean±SD. n=7 for each experimental group. * P <0.05. Statistical significance was determined by using unpaired t-test.

(**G**) qRT-PCR analysis of Nox2 (mdx veh, n=3; mdx JQ1, n=4), Nox4 (mdx veh, n=6; mdx JQ1, n=4), p47-phox (n=7 for each experimental group), p67-phox (n=6 for each experimental group) mRNAs in TAs cells of vehicle- and JQ1-treat 12-month-old mdx mice. Data are normalized against HPRT and expressed as the mean±SD. * $P < 0.05$, ** $P < 0.01$. Statistical significance was determined by using unpaired t-test.

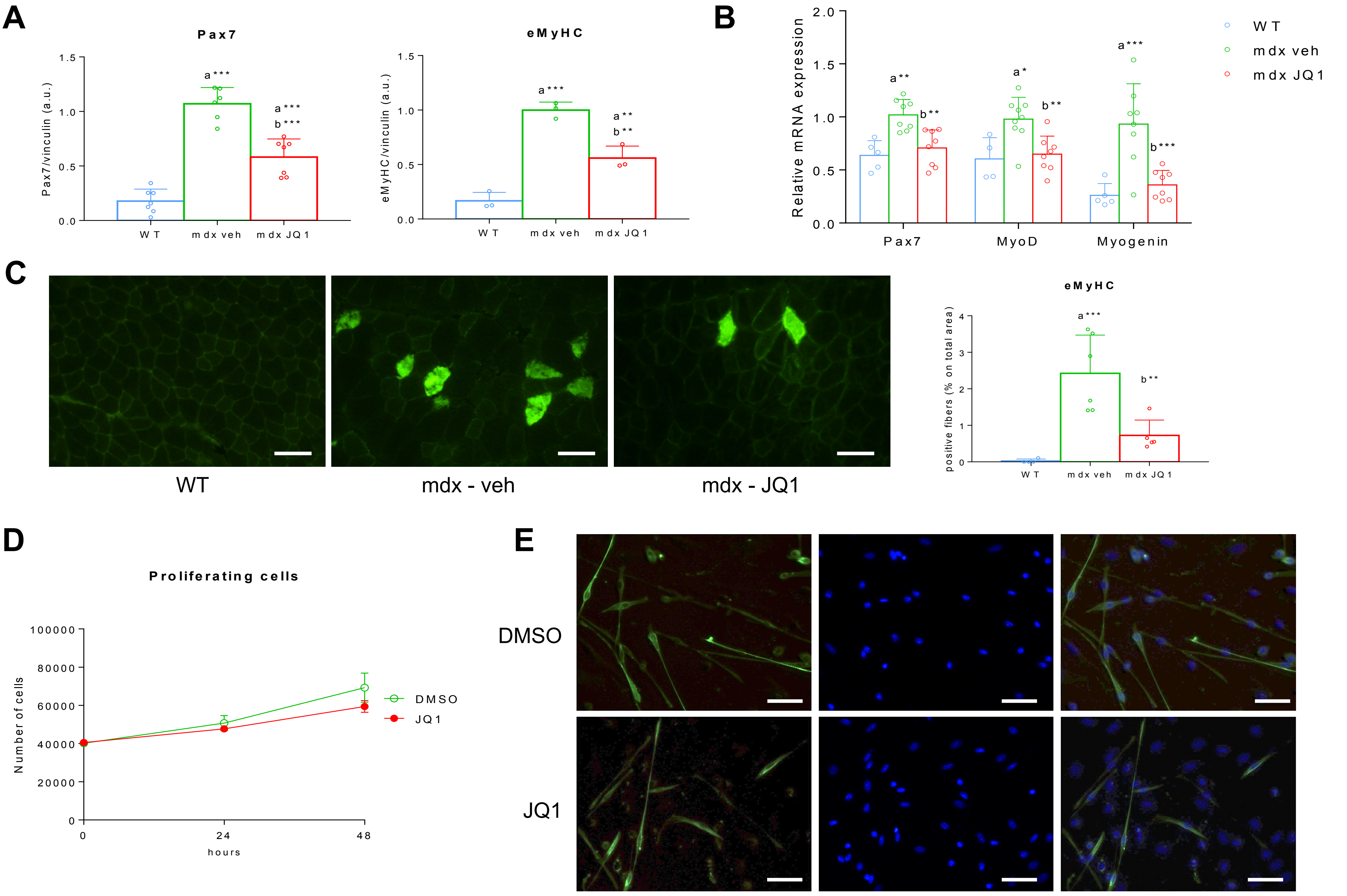

Supplementary Fig.12

Supplementary Figure 12. Regeneration markers decrease following JQ1 treatment.

(**A**) Signal quantification for pax7 (WT, n=7; mdx+veh, n=6; mdx+JQ1, n=7) and eMyHC (n=3 for each experimental group) of experiments shown in Fig. 6G. Data are expressed as the mean±SD.

(**B**) qRT-PCR analysis of Pax7 (WT, n=5; mdx, n=8; mdx+JQ1, n=9), MyoD (WT, n=4; mdx, n=9; mdx+JQ1, n=8) and Myogenin (WT, n=5; mdx, n=8; mdx+JQ1, n=8) mRNAs in TAs from control, vehicle-, JQ1-treated mice. Data are normalized against HPRT and expressed as the mean±SD.

(**C**) eMyHC immunofluorescence (green) in TA sections derived from WT, vehicle- and JQ1-treated mdx mice. WT, n=4; mdx+veh, n=6; mdx+JQ1, n=5. Data are expressed as the mean±SD. Scale bar: 50 μm. Right panel: percentage of eMyHC positive fibers per field.

In panels A-C, * denotes $P < 0.05$; ** denotes $P < 0.01$; *** denotes $P < 0.001$. Statistical significance was determined by using one-way ANOVA followed by Tukey's post hoc test. "a" indicates statistical significance compared to control; "b" indicates statistical significance compared to mdx.

(**D**) Satellite cells were isolated from 7-week-old mice and were treated for 48hr with 200nM JQ1. Cells were counted at 24 and 48 hr to evaluate cells growth. n=4. Data are expressed as the mean±SD. Statistical significance was determined by using one-way ANOVA followed by Tukey's post hoc test.

(**E**) Satellite cells were isolated from 7-week-old mice, expanded and switch to differentiation medium. Cells were treated either with DMSO or JQ1 (200nM) for 72 hr, fixed with 4% formaldehyde and immunofluorescence was performed with antibodies

raised against myosin heavy chain (MF20). Scale bar: 100 μm.

Supplementary Table 1. List of antibodies used in this study.

Antibody	*Cat. number*	*Company*	*Application and dilution*
Brd2	A302-583A	Bethyl	WB (1:1000), ChIP
Brd2	sc-393720	Santa Cruz	WB (1:1000)
Brd3	A302-368A	Bethyl	WB (1:1000)
Brd3	sc-81802	Santa Cruz	WB (1:1000)
Brd4	NBP118874/A301985A100	NovusBio/Bethyl	WB(1:1000), ChIP
Brd4	sc-27976	Santa Cruz	WB (1:1000)
Vinculina	V9264	SIGMA	WB (1:10000)
GAPDH	sc-32233	Santa Cruz	WB (1:3000)
Nox2	ab12906	Abcam	WB (1:1000)
P67-phox	15551-1-ap	Proteintech	WB (1:1000)
Sirt1	07-131	Millipore	WB (1:1000)
AMPKα1/2	sc-25792	Santa Cruz	WB (1:1000)
p-AMPKα1/2 (Thr 172)	sc-33524	Santa Cruz	WB (1:1000)
LC3b	L7543	SIGMA	WB (1:2000)
P62	sc-28359	Santa Cruz	WB (1:1000)
ULK1(H240)	sc-33182	Santa Cruz	WB (1:1000)
p-ULK 1 (Ser 556)	T A310925	Origene	WB (1:300)
Akt1/2/3 (H136)	sc-8312	Santa Cruz	WB (1:1000)
p-Akt1/2/3 (Ser 473)	sc-7985-R	Santa Cruz	WB (1:1000)
mTOR	sc-8319	Santa Cruz	WB (1:1000)
p-mTOR (Ser2448)	sc-293133	Santa Cruz	WB (1:500)
p70S6k	sc-230	Santa Cruz	WB (1:1000)
p-p70S6k (Thr389)	sc-11759	Santa Cruz	WB (1:1000)
AcetylH3K9	06-942	Millipore	WB (1:1000)
AcetylH3K14	07-353	Millipore	WB (1:1000)
Src	05-184	Millipore	WB (1:1000)
p-Src (p Y^{418})	44-660	Biosource	WB (1:1000)
F4/80	sc-377009	Santa Cruz	WB (1:1000), IF (1:50)
CD45	sc-52490	Santa Cruz	WB (1:1000), IF (1:50)
PAX7	sc-81648	Santa Cruz	WB (1:1000), IF

			(1:30)
eMyHC	BF-45	Dshb	WB (1:200)
NF-KB p65	SC-372	Santa Cruz	WB (1:2000)
p-NFKB p65 (Ser536)	sc-33020	Santa Cruz	WB (1:1000)
IL-6 (D5W4V)	#12912S	Cell Signalling	WB (1:1000)
Casp3	sc-7148	Santa Cruz	WB (1:1000)
8OH-dG	sc-66036	Santa Cruz	IF (1:1000) in BSA 3%
Tubulin 6		dr. J. Ervasti	WB (1:1000)
Tubulin alpha	T9026	Sigma	WB (1:1000)
8OH-dG	sc-66036	Santa Cruz	IF (1:1000) in BSA 3%
detyrosinated Tubulin	AB3201	Millipore	WB (1:1000)
Alexa 488 goat-anti rabbit IgG	A11008	Thermo Fisher	IF (1:1000)

Supplementary Table 2. List of primers used in quantitative RT-PCR analysis

Gene	*Sequence*
mNox2	GAC CCA GAT GCA GGA AAG GAA<br>TCA TGG TGC ACA GCA AAG TGA T
mNox4	CGA GAC TTT TCA TTG GGC GTC CTC<br>TAG AAC TGG GTC CAC AGC AGA AAA
mp47-phox	GAG GTT GGG TCC CTG CAT<br>GCT TTG ATG GTT ACA TAC GGT TC
mp67-phox	GCT GCG TGA ACA CTA TCC TG<br>AGG TCG TAC TTC TCC ATT CTG
mOptn	GAG CAG ACA GAG AGA AGG AGG<br>GTG TGTGCC TCT TGA AGC TC
mAtg2A	TCA TGG GAC TCC TAA TCGGC<br>GCA GCT GGT TCT TAC GGA TG
mAtg9B	CCG GGT ACA ACA AGA TGC AG<br>CTT CTG GCA GAG GGG ATG TAC
mSqstm1	GCT CCA CCA GAA GAT CCC AA<br>TTT CTG GGG TAG TGG GTG TC
mBrd4	CCT CCC AGT GTG CCC CTT CTT<br>CTG AGT CGG AGA GCA CCA GCG
mBrd2	CCC ACC CAT CAG TCA TCT CT<br>GGG GTG GTA GTA TCC GCT TT
mBrd3	CCC CTG TAC CAA CCA TCA CT<br>GTG TCT GCT TTC CGC TTC AC
mGAPDH	AAC ATC AAA TGG GGT GAG GCC<br>GTT GTC ATG GAT GAC CTT GGC
mHPRT	GGC CAG ACT TTG TTG GAT TTG<br>TGC GCT CAT CTT AGG CTT TGT
mFibronectin	GCT GGA TGA TGG TGG ACT GT<br>CTC GGT TGT CCT TCT TGC TC
mIL6	GCC AGA GTC CTT CAG AGA GA<br>TGG TCC TTA GCC ACT CCT TC
mSOCS3	GCA AGC TGC AGG AGA GCG GAT T<br>AAG AAG TGG CGC TGG TCC GA
mTNF-alpha	CTG TAG CCC ACG TCG TAG C<br>TTG AGA TCC ATG CCG TTG
mPax7	AGG ACG ACG AGG AAG GAG ACA<br>TCA TCC AGA CGG TTC CCT T

mMyoD	GGC TAC GAC ACC GCC TAC TA<br>GTG GAG ATG CGC TCC ACT AT
mMyogenin	CTA CAG GCC TTG CTC AGC TC<br>GCT GTG GGA GTT GCA TTC AC
mHmox1	GTC AAG CAC AGG GTG ACA GA<br>CTG CAG CTC CTC AAA CAG CT
mGclm	TGT GTG ATG CCA CCA GAT TTG<br>ATG CTT TCT TGA AGA GCT TCC T
mGclc	ATT GTC GCT GGG GAG TGA TT<br>TAT CTA TTG AGT CAT ACC GAG A
hNox2	GTA TCC ATA TCC GCA TCG TTG G<br>ATC ACC ACC TCA TAG CTG AAC A
hNox4	ACC GAA CCA GCT CTC AGA ATA T<br>GTC CAG AAA TCC AAA GCC AAG T
hp47-phox	CCA CCT CCT CGA CTT CTT CAA<br>GTC TTC TCG TAG TTG GCA ATGG
hp67-phox	CTG GAG TTT CAG GAA GGG GAT A<br>CTA GAC TTC TCT CCG AGT GCT T
hGAPDH	GCC TCA AGA TCA TCA GCA ATG C<br>CCA CGA TAC CAA AGT TGT CAT GG
mAtg8	GCA GGA GAC ATT CGA ACA GA<br>GAG GCT CCT GAA AGT CCA AG
mLC3b	GTC CGA GAA GAC CTT CAA GC<br>AAG CGC CGT CTG ATT ATC TT
mCol1a1	CAT GTT CAG CTT TGT GGA CCT<br>GCA GCT GAC TTC AGG GAT GT

Supplementary Table 3. List of primers used in ChIP analysis

Gene	*Sequence*
mNox2	GCT TCA GTG AGG ACC CAA TC<br>CCA CTT TTC CAT CAT CCA TGT
mNox4	ATA GGG AAG GCT AAT ACA CAA AAG G<br>CCA AAA TGA CAG AAA GAA AAG AAA A
mp47-phox	AGA ATT TTT GAG CTT CAA CAG AGT G<br>GGT GGT TCA GAA CTG TAG AGA GG
mp67-phox	CCA GCG CTA AGA TTA GAA AGG AT<br>CTC ACA GCC TAC AGA GTC CAA GT

Supplementary Table 4. List of siRNA used in transfection

siRNA	*Provider*	*Sequence*
siBrd2	Dharmacon	L-043404-00-0005
siBrd3	Eurofins	UCC GGU UAA UGU UCU CGA A<br>UUC GAG AAC AUU AAC CGG A
siBrd4	Invitrogen	ACG UCC AGC CUA AGA AGC AGG UAA A<br>UUU ACC UGC UUC UUA GGC UGG ACG U
siScramble	Eurofins	GCG UUG CUC GGA UCA GAA A<br>UUU CUG AUC CGA GCA ACG C

Supplementary Table 5. Donors for immortalized DMD muscle cells.

Dystrophin gene mutation	*Reference*	*Muscle*	*Age*	*Sex*
mutation stop exon 59:<br>c.8713C>T, p.Arg2905X	1	Quadriceps	11 y	male
Del 45-50	2	Paravertebral	14 y	male
Duplication exon 10-11	3	Dorsal	14 y	male

Supplementary Table 6. Human tissues

Specimen	*Reference*	*Muscle*	*Age*	*Sex*

Control	24522	paravertebral	17 y	male
Control	24400	paravertebral	16 y	male
DMD	30739	paravertebral	15 y	male
DMD	27481	paravertebral	14 y	male
DMD	31218	paravertebral	13 y	male
DMD	25832	paravertebral	12 y	male

Supplementary references

1 Segatto, M. *et al.* Simvastatin treatment highlights a new role for the isoprenoid/cholesterol biosynthetic pathway in the modulation of emotional reactivity and cognitive performance in rats. *Neuropsychopharmacology* **39**, 841-854, doi:10.1038/npp.2013.284 (2014).

2 Proserpio, V., Fittipaldi, R., Ryall, J. G., Sartorelli, V. & Caretti, G. The methyltransferase SMYD3 mediates the recruitment of transcriptional cofactors at the myostatin and c-Met genes and regulates skeletal muscle atrophy. *Genes Dev* **27**, 1299-1312, doi:10.1101/gad.217240.113 (2013).

3 Fenizia, C. *et al.* SMYD3 promotes the epithelial-mesenchymal transition in breast cancer. *Nucleic Acids Res*, doi:10.1093/nar/gky1221 (2018).

4 Khairallah, R. J. *et al.* Microtubules underlie dysfunction in duchenne muscular dystrophy. *Sci Signal* **5**, ra56, doi:10.1126/scisignal.2002829 (2012).
